# Case of Scrofulous Ulcers of the Leg, with Injury of the Ankle Joint—Amputation for

**Published:** 1861

**Authors:** L. B. Brown

**Affiliations:** Iroquois, Ills.


### CASE OF SCROFULOUS ULCERS OF THE LEG,—WITH INJURY OF THE ANKLE JOINT.—AMPUTATION FOR.

By L. B. BROWN, M. D., Iroquois, Ills.

Editors of the Chicago Medical Journal.—Henry Enshins, aged 66 years, a resident of Iroquois, Illinois, has suffered at intervals for several years, from scrofulous ulcers of the left leg; but never compelled to discontinue his attendance to business till October 1860, when he received an injury of the ankle joint jumping from a carriage. The foot and ankle became very painful and somewhat swollen.—Domestic medicine was brought into requisition, and faithfully applied for nearly two months, when a large sloughing ulcer presented at the heel, and several small ulcers about the ankle joint and top of the foot. A physician was now called, and constitutional treatment brought to bear on the sore heel, but without relief. I visited the patient for the first time, in February, 1861. I found him in bed, greatly emaciated, having a voracious appetite, diarrhœa, and suffering from pain in the foot and ankle. The foot and ankle presented a horrible appearance, studded with fistulous openings, discharging a large amount of offensive matter; the tendo Achillis, muscles of the heel, and posterior ligaments of the ankle joint, were completely destroyed; leaving the oscalcis, tibia, and fibula, bare. I told the patient and family that he could not live, in the condition he was in; I spoke of amputation as the only chance for relief, but did not recommend it, owing to the extreme exhaustion. March 2d. Visited the patient to-day in company with Drs. Barry, Fowler and Dunn. After stating to the patient and family the certainty of death,—without, and the chance of recovery with an operation, consent was given, and the operation was performed. Chloroform was given as an asthetic, the limb amputated in its upper third, just below the tuberasity of the tibia, by the flap method; very little blood was lost during the operation. The flaps were brought together by interrupted sutuses and strips of adhesive plaster, the usual dressings applied, and the whole confined with a roller bandage. The patient placed in bed, and brandy and opium given during the remainder of the day and night.

March 3d. Patient rested a part of last night after taking one half gr. Morphine. Pulse 80, no pain, except an occasional twinge in the stump; stump not disturbed. Ordered brandy and quinine during the day, anodyne at night.

March 4th. No change of importance during the last 24 hours. Continue the same treatment.

March 5th. Rested very well last night; no pain in the stump. Pulse 80, the flaps of the wound lying against each other without any adhesion, or evidence of inflamation; slight evidence of suppuration. Same dressings re-applied, and ordered in addition to brandy and quinine, beef-tea.

March 6th. Pulse 80, suppuration of the stump; discharging unhealthy pus. Ordered the stump thoroughly washed with tinct. myrry, and covered with pulv. charcoal. Continue same constitutional treatment.

March 7th. Suppuration going on rapidly; stitches give way to-day: same constitutional and local treatment as yesterday.

March 9th. Patient exhibits signs of exhaustion; appetite failing, no pain; surface covered with cold perspiration; discharge from the wound copious, and offensive; the sown extremity of the tibia exposed. Ordered the surface sponged with sal. chlo. sodium; the same constitutional and local treatment as before, with increase of brandy.

March 12th. Patient thirsty, skin hot; pulse 90; stump inflamed for the first time since the operation, and somewhat painful; discharge from the wound not as copious. Continue the use of brandy, quinine and beef-tea.

March 15th. Patient improving; discharge from the stump greatly diminished. Some appearance of healthy granulation. Continue the use of brandy and quinine, together with a nutritious diet.

April 15th. The daily notes of the case since March 15th contain nothing of importance. The wound, owing to the impaired condition of the system, healed slowly. The patient is improving in general health; appetite good; diarrhœa stopped; and the wound nearly cicatrised.

The above case, I think, is one of interest, considering the advanced age, and the extreme debility of the patient at the time of the operation; I do not offer it, however, as an anomaly; but, from the fact that I have been censured (for operating) by a few meddlesome people, some of whom claim to belong to the Medical Profession.

